# Statistical shape modeling of shape variability of the human distal tibia: implication for implant design of the tibial component for total ankle replacement

**DOI:** 10.3389/fbioe.2025.1504897

**Published:** 2025-02-27

**Authors:** Jian Yu, Chengke Li, Jinyang Lyu, Shengxuan Cao, Chao Zhang, Xin Ma, Dahang Zhao

**Affiliations:** ^1^ Department of Orthopedics, Shanghai Sixth People’s Hospital Affiliated to Shanghai Jiao Tong University School of Medicine, Shanghai, China; ^2^ Department of Hand and Foot Microsurgery, Shunde Hospital Affiliated to Jinan University, Foshan, Guangdong, China; ^3^ Department of Orthopedics, Huashan Hospital, Fudan University, Shanghai, China; ^4^ Department of Orthopedics, Ruijin Hospital Affiliated to Shanghai Jiao Tong University School of Medicine, Shanghai, China

**Keywords:** distal tibial morphology, human anatomy, statistical shape modeling, total ankle replacement, implant design

## Abstract

**Introduction:**

Understanding the morphological variability of the distal tibia can help design tibial components of total ankle implants. This study aimed to assess the shape variability of the distal tibial bone, utilizing the statistical shape modeling (SSM) technique.

**Methods:**

A total of 229 tibial bones were analyzed through CT scans to develop SSM models. Principal component analysis (PCA) was employed to characterize shape variation across the male, female, and overall groups. The geometric parameters of the resected surfaces at the 10-mm level above the distal tibial articular surface were compared.

**Results:**

The first seven principal component analysis (PCA) modes accounted for approximately 24.9%–40% of the shape variation, totaling 71.5%–75.6%. Considerable variabilities were observed among these three groups and all principal modes of variation. Notably, the male tibia had a bigger medial malleolus, anterior part of the fibular notch, and posterior malleolus. In the 10-mm resection surface of the distal tibia, anterior–posterior and medial–lateral distances were the main sources of variation. In addition, variations were frequently detected at both the anterior and posterior corners of the fibular notch in the resection surface of the distal tibia.

**Conclusion:**

The SSM technique has been shown to be an effective method in finding mean shape and principal variability. Size plays a crucial role in both inter- and intra-groups, and morphological differences vary across different sizes. Therefore, these considerations should be taken into account while designing tibial components for total ankle implants.

## Introduction

Total ankle arthroplasty (TAA) is an effective procedure to treat end-stage ankle arthritis (Raikin et al., 2014). The implant survival rate for TAA is improving (Gougoulias et al., 2010; Daniels et al., 2014), yet it is not comparable to that of total knee or hip arthroplasty (Lee et al., 2018; Clough et al., 2019; Mäkelä et al., 2014; Victor et al., 2014). During TAA, the damaged distal tibia was replaced by a metallic artificial implant. Novel tibial components of total ankle implants, considering maximum distal tibial coverage (Gross et al., 2018), require detailed measurements of the anatomy of distal tibia and its resection surface. In addition, understanding the three-dimensional (3D) shape variability of the distal tibia can help identify the shape morphological similarities and differences in patients with pathological changes in the tibia, such as the tibial fracture, anterior impingement, and osteochondral lesions of the distal tibia (Mitchell et al., 2019; Blom et al., 2019).

However, the shape of the distal tibia is complex and not fully understood, with size and gender differences (Claassen et al., 2019; Ataoğlu et al., 2020). Current clinical evaluation methods of the measurement of the bone rely on plain radiographic images or images from computed tomography (CT) or magnetic resonance imaging (MRI). After the selection of feature points on the anatomic landmark of the images, distances, angles, or areas can be measured to describe the bone anatomy (Yu et al., 2022; Nguyen et al., 2020; Kuo et al., 2016). However, only a limited amount of image information is utilized, and selection bias might exist when choosing feature points. Statistical shape modeling (SSM) serves as a robust analytical tool for analyzing anatomical data by constructing a mean shape and several variations from a collection of medical images (Heimann and Meinzer, 2009; Lenz et al., 2021). Although several studies (Bredbenner et al., 2010; Audenaert et al., 2019; Peiffer et al., 2022; Nelson et al., 2017; Gabrielli et al., 2020) focused on the tibia or ankle joint, which included morphological studies of the distal tibia, to the best of our knowledge, we found no articles specifically studying the SSM of distal tibia and reporting the shape change at the distal tibial resection surface.

In this study, we aimed to qualitatively evaluate the shape variability of the distal tibial bone using the SSM method. Male and female tibiae were registered separately and used to generate two separate SSMs, thereby producing two sex-specific mean tibia shapes that were then analyzed for gender differences between SSM models. The resection surfaces in the distal tibia for total ankle replacement were compared among different shape modes.

## Materials and methods

With the Institutional Review Board approval, the computed tomography (CT) scan data on 123 healthy Chinese participants (59 females and 64 males, 106 participants contributed both ankles; 23.78 ± 3.19 years of age, 168.52 ± 7.70 cm of height, and 63.08 ± 13.42 kg of body weight; body mass index: 22.06 ± 3.50) from previous studies were used (Yu et al., 2023; Wang et al., 2023). Two orthopedic surgeons independently evaluated all CT images (scanned by Brilliance iCT, Philips, Cleveland, U.S. with 120 kV of voltage, 250 mA of current, 0.67 mm of slice thickness, and 512 × 512 pixels of matrix) (Yu et al., 2022) to exclude previous trauma, severe deformity, or degenerative changes in the ankle, such as ankle arthritis and osteochondral lesions of the distal tibia.

### 3D reconstruction

A total of 229 3D models of tibia were reconstructed in Mimics (Materialise NV, Belgium) from the Digital Imaging and Communications in Medicine (DICOM) file of CT images. All right tibiae were mirrored and grouped together with the left tibia in 3-matic Medical (Materialise NV, Belgium). A parallel cut was made at 25 mm above the distal tibial articular surface of each tibial bone to create the distal tibia (Hvid et al., 1985). A sensitivity analysis of the selection on the articular surface of the distal tibia for plane fitting on one of the subjects was performed (See Supplementary Figure S1; Supplementary Table S1 of the supplementary document).

### Statistical shape modeling

An open-source SSM (ShapeWorks, University of Utah, Salt Lake City, UT, United States) was used for the statistical shape modeling of the distal tibia (Cates et al., 2017). Each tibia was first aligned to a randomly chosen “master” tibia using the iterative closest point method. Then, surface meshes were converted to volumetric datasets in the form of distance transforms. ShapeWorks software used 1,024 anatomical landmarks to represent each distal tibia, and the correspondence landmark locations of all tibiae can be analyzed for mean shapes and shape variations.

### Analysis

**FIGURE 1:**
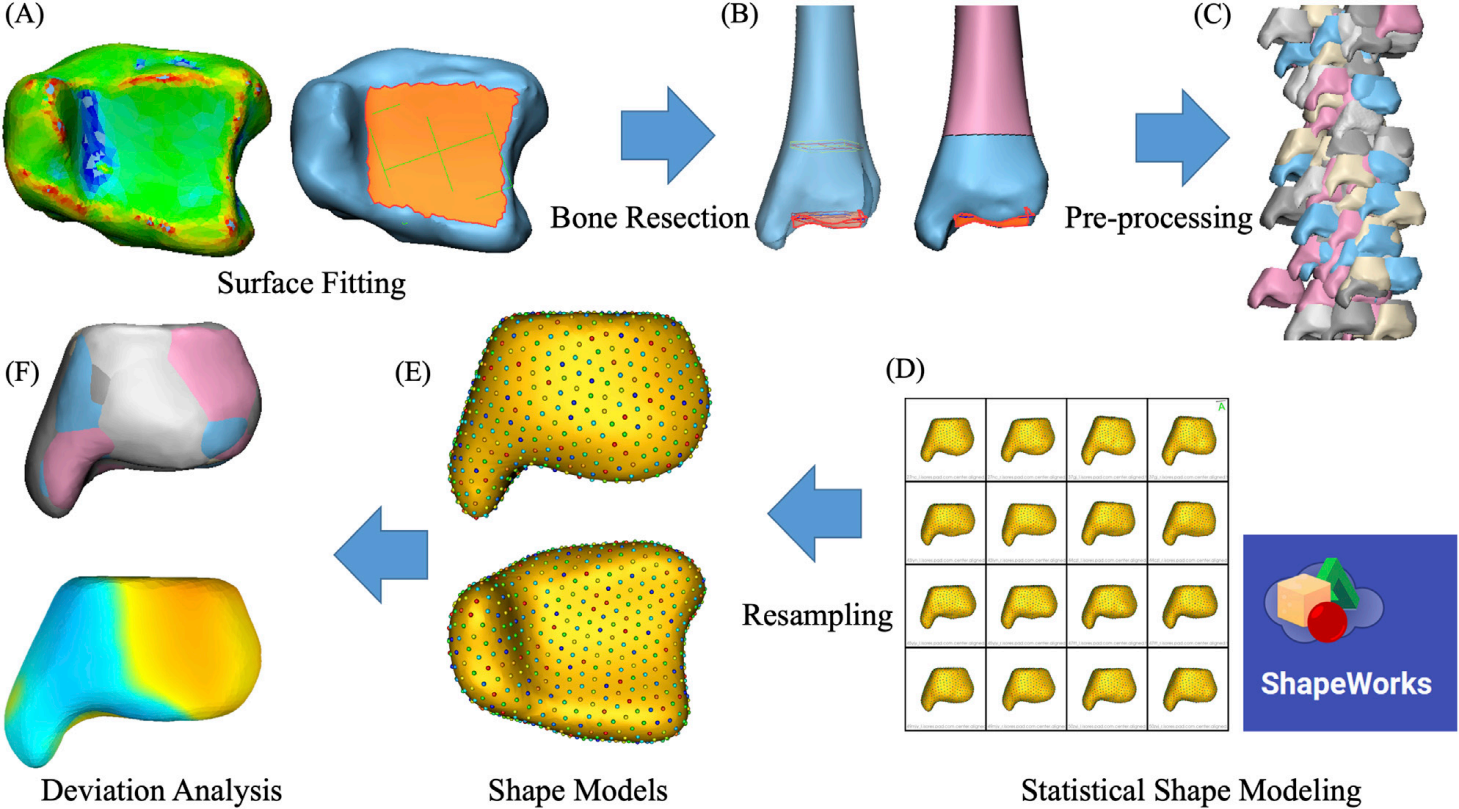
Flowchart of image and model processing. **(A)** The distal articular surface of the tibia was fitted with a datum plane. **(B)** Distal tibia was created by bone resection. **(C)** Model pre-processing process. **(D)** Statistical shape modeling with the ShapeWorks. **(E)** Shape models with correspondence landmarks. **(F)** 3D models from the principal component analysis and deviation analysis.

Principal component analysis (PCA) can reduce high-dimensional SSM correspondence data and yield non-zero eigenvalues that characterize the amount of variance. Each uncorrelated dimension of variation was defined as “modes” based on the order of the eigenvalues. For each significant mode of tibia, the mean and ±3 standard deviations (SD) of the surface model were exported in Geomagic Studio 2013 (Geomagic, Morrisville, North Carolina, United States). Deviation analysis was performed to visualize anatomical differences within a mode of variation, in which the mean tibial bone was used as the reference model, while the ±3 SD tibia was used as a target model. The SSM process is presented in Figure 1.

### Post-processing

The recommended tibial bone resection level for total ankle replacement ranges from 5 mm to 11 mm among different implant systems (Yu et al., 2020). To maintain consistency and create a resection surface for total ankle replacement, the tibial bone was resected at 10-mm level superior to tibial plafond with the protection of medial malleolus under the guidance of senior foot and ankle surgeons (Yu et al., 2022).

**FIGURE 2:**
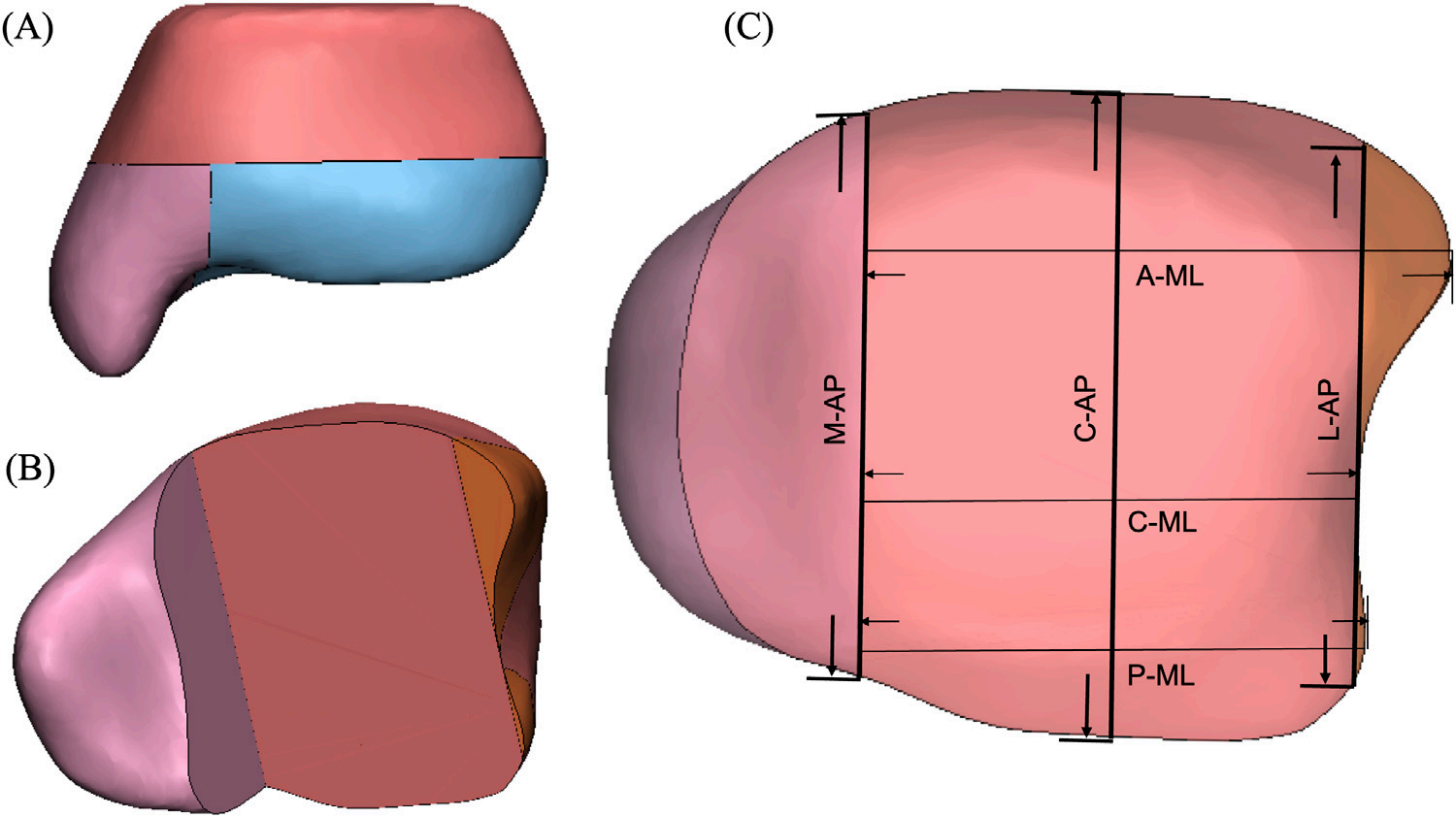
Schematic representation of the distal tibial resected surface showing the measurement methods used in the CT analysis. **(A)** Distal tibial cut was made at 10-mm level superior to tibial plafond with the protection of the medial malleolus. **(B)** Perspective view of the distal tibia after the removal of the 10-mm bone fragment of the distal tibia. **(C)** Two-dimensional illustration of the geometric parameters of the 10-mm resection surface of the distal tibia. M-AP, C-AP, and L-AP represented the anterior–posterior distances, while A-ML, C-ML, and P-ML represented the medial–lateral distances.

At the 10-mm resection surface, the medial anterior–posterior (M-AP) dimension was taken as the medial largest anteroposterior length. The lateral anterior–posterior (L-AP) dimension was taken as the length of the line drawn parallel to the M-AP and passing though the medial-most point in the fibula notch of the distal tibia. The C-AP is the anterior–posterior distance of the surface along the middle line of M-AP and L-AP. Perpendicular to M-AP, anterior medial–lateral (A-ML) and posterior medial–lateral (P-ML) dimensions were taken as the anterior and posterior longest mediolateral lengths of the resected distal tibial surface, respectively, while C-ML represented the shortest mediolateral length of the resected distal tibial surface (Figure 2C). These geometric parameters of the 10-mm resection surface of the distal tibia were compared for each mode of variation.

## Results

### Tibial shape variation for the overall, male, and female groups

**FIGURE 3:**
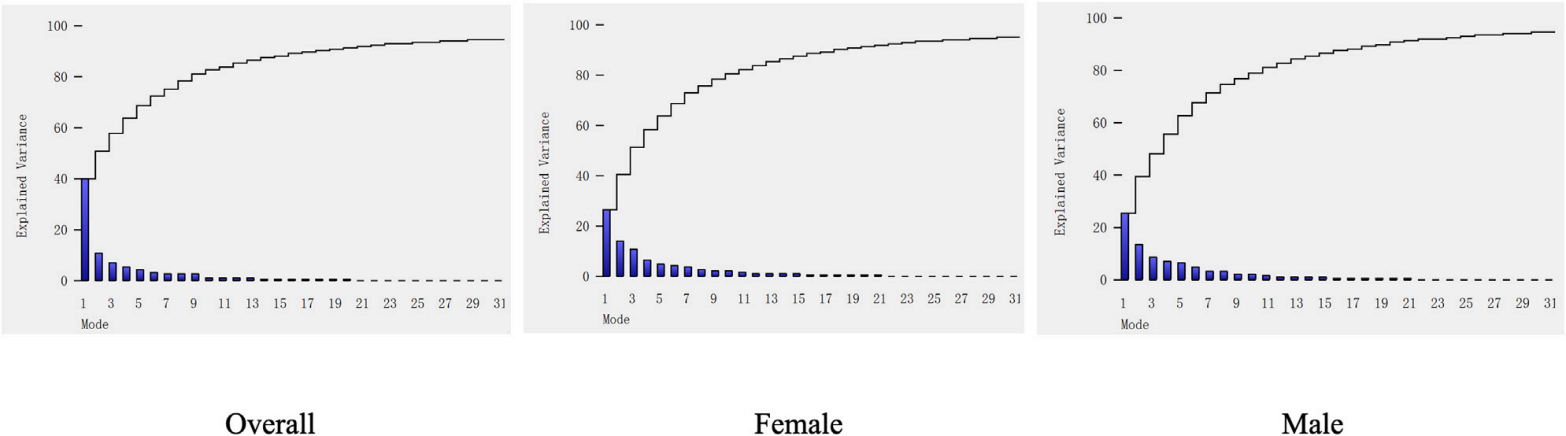
Cumulative shape variation in significant PCA modes of the overall, male, and female groups. PCA modes are ordered based on the associated variance (bar), which determined the cumulative shape variation (curve).

Seven PCA components for the overall tibiae represented 40.0%, 11.2%, 7.1%, 5.9%, 4.6%, 3.8%, and 3.1% of the overall variation, respectively, which contributed to a cumulative total of 75.6% of the overall shape variation. For the female group, the first seven modes accounted for 26.5%, 14.5%, 10.9%, 6.8%, 5.4%, 4.8%, and 4.1% of the overall tibial variation, respectively, collectively representing 73.0% of the female shape variation. For male groups, the first seven modes represented 25.9%, 13.7%, 8.8%, 7.4%, 7.0%, 5.2%, and 3.6% of the overall tibial variation, respectively, representing 71.5% of the male shape variation in total (see Figures 3).

**FIGURE 4:**
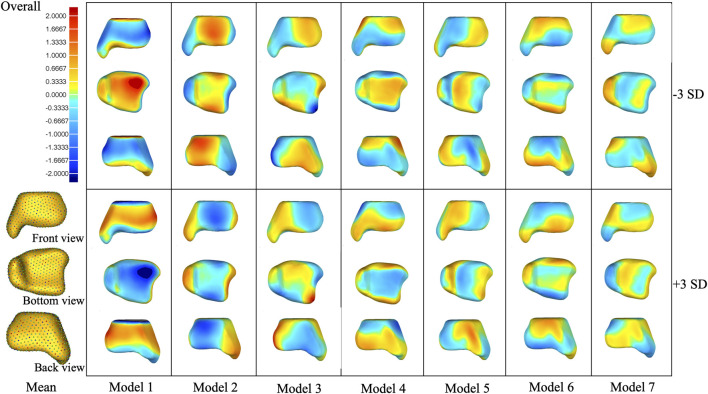
Shape variation was captured in PCA modes 1–7 of the overall group. Within each mode, shape variations are shown at ±3 SD from the mean shape. Contour plots identify areas of deviation with respect to the mean shape.

**FIGURE 5:**
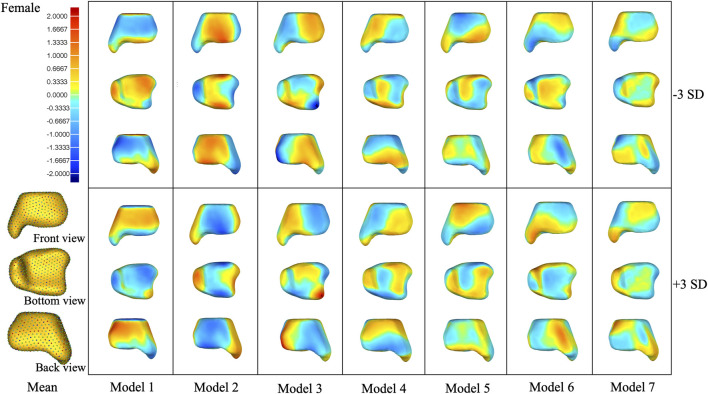
Shape variation was captured in PCA modes 1–7 of the female group. Within each mode, shape variations are shown at ±3 SD from the mean shape. Contour plots identify areas of deviation with respect to the mean shape.

**FIGURE 6:**
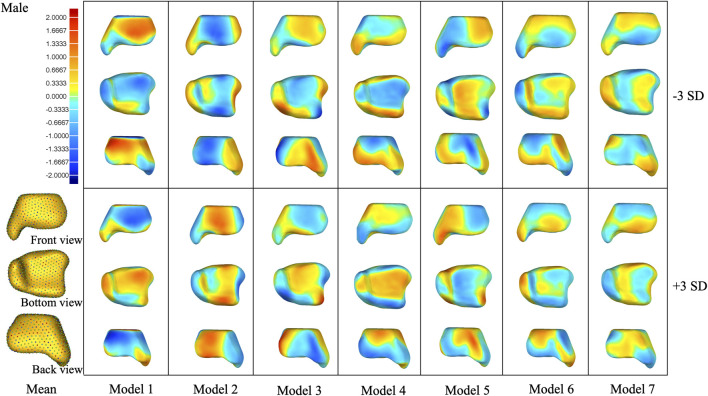
Shape variation was captured in PCA modes 1–7 of the male group. Within each mode, shape variations are shown at ±3 SD from the mean shape. Contour plots identify areas of deviation with respect to the mean shape.

Deviation analyses of the shape variations (+/−3 SD) with respect to their mean shapes are presented in Figures 4–6 and Supplementary Table S2 of the supplementary document.

In the overall group, the first mode of variation showed a remarkable variation in the tibia from inner–outer and top–bottom directions changing from a short and thick shape to a long and thin shape. Differences in the second mode of variation included a variation in the tibia at the anterior–posterior and medial–lateral dimensions. The third mode of variation showed a variation in the tibia from anterior–lateral to posterior–medial directions. The fourth mode of variation exhibited a variation in the tibia from anterior-bottom to posterior-top directions. The fifth mode of variation described a variation in anterior–posterior lengths between a wider medial side and narrow lateral side to the opposite with the associated thickening of the medial malleolus. The sixth mode of variation was a combination of a variation in the anterior–posterior and up–down directions. The seventh mode of variation includes small changes at the tip of the medial malleolus, the anterior lateral, and posterior edge of the tibia.

The seven modes of variation in both the female and male groups shared the same trend with the overall group. Small differences were presented in the fourth, fifth, and sixth modes of variation between the female and overall groups. The fourth mode of variation in the female group exhibited a variation in the tibia from posterior–medial to anterior–lateral directions. The fifth mode of variation described a variation at the anterior lateral and posterior edges of the tibia. The sixth mode of variation showed small changes at the anterior and posterior edges of the medial malleolus. Differences between the male and overall groups were noticed in the fourth modes of variation, where the male group displayed a combination of a variation in up–down directions and around the upper and bottom edges.

### Gender differences between mean models

**FIGURE 7:**
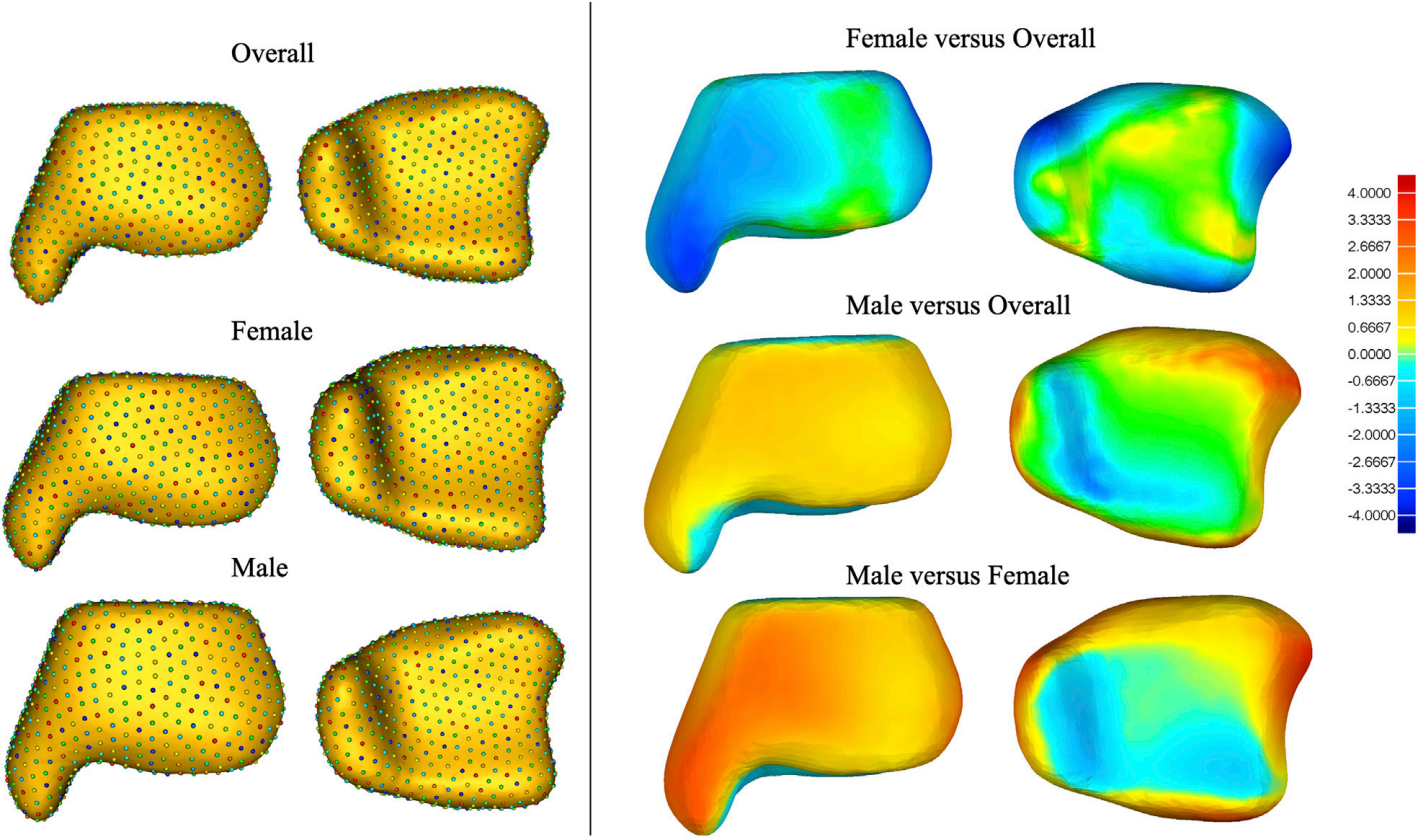
Shape deviation among mean shapes of the overall, male, and female tibiae.

Shape deviations of mean shapes among the three groups are presented in Figure 7 and in Supplementary Table S3 of the supplementary document. It was noted that the mean shape of the male tibia generally has a large size than the female tibia, especially around the medial malleolus, anterior part of the fibular notch, and posterior malleolus.

### Curve variation in the resection surface of PCA modes 1–7 of the overall group

**FIGURE 8:**
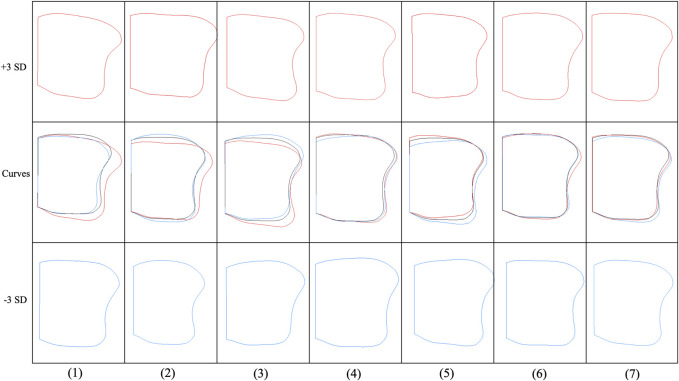
Curve variation in the resection surface of PCA modes 1–7 of the overall group. Within each mode, curve variations are shown at ±3 SD from the mean shape (the red curves represent +3 SD models, the black curves represent mean models, and the blue curves represent −3 SD models).

The first mode of variation showed a remarkable variation in size along the anterior–medial to posterior–lateral directions. The +3 SD model of the first mode generally has a bigger anterior–posterior and medial–lateral distance than the −3 SD model and is rotated clockwise (Figure 8; Table 1). However, the L-APs of +3 or −3 SD models of the first mode were smaller than those of the mean model, indicating that the variation models were reducing in anterior–posterior distance on the lateral side. Differences in the second mode of variation included a variation from the medial–lateral direction. The +3 SD model of the second mode was wider but thinner than the −3 SD model (the +3 SD model of the second mode has smaller M-AP, C-AP, and L-AP but larger A-ML, C-ML, and P-ML).

**TABLE 1:** Geometric parameters of the 10-mm resection surface of the distal tibia.

Overall group	M-AP (mm)	C-AP (mm)	L-AP (mm)	A-ML (mm)	C-ML (mm)	P-ML (mm)
Mean	31.81	36.26	30.33	32.86	27.72	28.38
Mode 1+3SD	31.83	38.08	29.77	37.24	29.67	29.72
Mode 1−3SD	30.79	34.46	28.76	31.77	26.17	26.54
Mode 2+3SD	30.11	33.34	25.66	35.95	28.24	29.25
Mode 2−3SD	31.51	38.73	33.27	33.01	27.29	28.37
Mode 3+3SD	30.68	35.81	32.93	33.66	28.70	30.46
Mode 3−3SD	31.45	36.84	29.33	34.39	27.88	27.88
Mode 4+3SD	32.15	36.56	31.10	33.99	28.78	29.68
Mode 4−3SD	30.1	35.88	29.77	33.83	28.84	29.16
Mode 5+3SD	33.22	35.98	28.64	32.67	27.89	28.41
Mode 5−3SD	29.14	35.97	32.26	34.29	28.8	29.46
Mode 6+3SD	31.15	36.77	30.06	34.23	28.02	28.09
Mode 6−3SD	31.76	35.44	30.74	32.64	27.99	28.94
Mode 7+3SD	32.17	36.31	28.28	33.46	27.63	27.68
Mode 7−3SD	30.92	36.34	20.66	34.14	28.43	28.99

The third mode of variation showed an obvious variation at both the anterior and posterior corners of the fibular notch. The fourth mode of variation exhibited a small variation at the anterior–medial edge and the lateral side of the resection surface. The fifth mode of variation described a small variation in the tibia at the anterior–medial, anterior–lateral, and posterior–lateral edge. Small variations in sixth and seventh modes are located around the lateral edge of the resection surfaces.

## Discussion

In this study, we developed SSMs of the distal tibia from CT images of 229 tibiae. Considerable variabilities were observed among these three groups and all principal modes of variation, highlighting the complexity of the 3D shape of the distal tibia, which cannot be clearly represented by two-dimensional (2D) radiographs or described using 2D measurements. The first seven principal component analysis modes accounted for approximately 24.9%–40% of the shape variation, totaling 71.5%–75.6%. Although 75.6% from first seven principal components may not fully explain the morphology of the distal tibia, seven modes of morphological variation are enough for implant design of the tibial component of the total ankle implant. Although ignorance of other principal components may result in the loss of some information, it may also prevent noise and the interpretation of random variation in the data. Future studies should further include the quantitative method, such as parallel analysis, to determine the significance of each mode.

Notably, the overall groups have a higher explained variance in the first PCA mode or the cumulative explained variance for the first seven PCA modes. It can be explained that the variances caused by the difference in the mean shape between males and females are much larger than the within-group variation, resulting in a significantly higher explained variance by the first PCA mode in the overall group. Gender differences revealed substantial size variation between the mean shape of male and female tibiae, especially in the medial malleolus and the anterior part of the fibular notch. Sex-specific implants with different shapes might play an important role in future implant designs. Chinese female patients, in particular, frequently experience issues with undersized implant and mismatch, which requires further investigation on these anatomical data. Of course, future studies should further investigate the shape variance, following size normalization in all distal tibiae.

**FIGURE 9:**
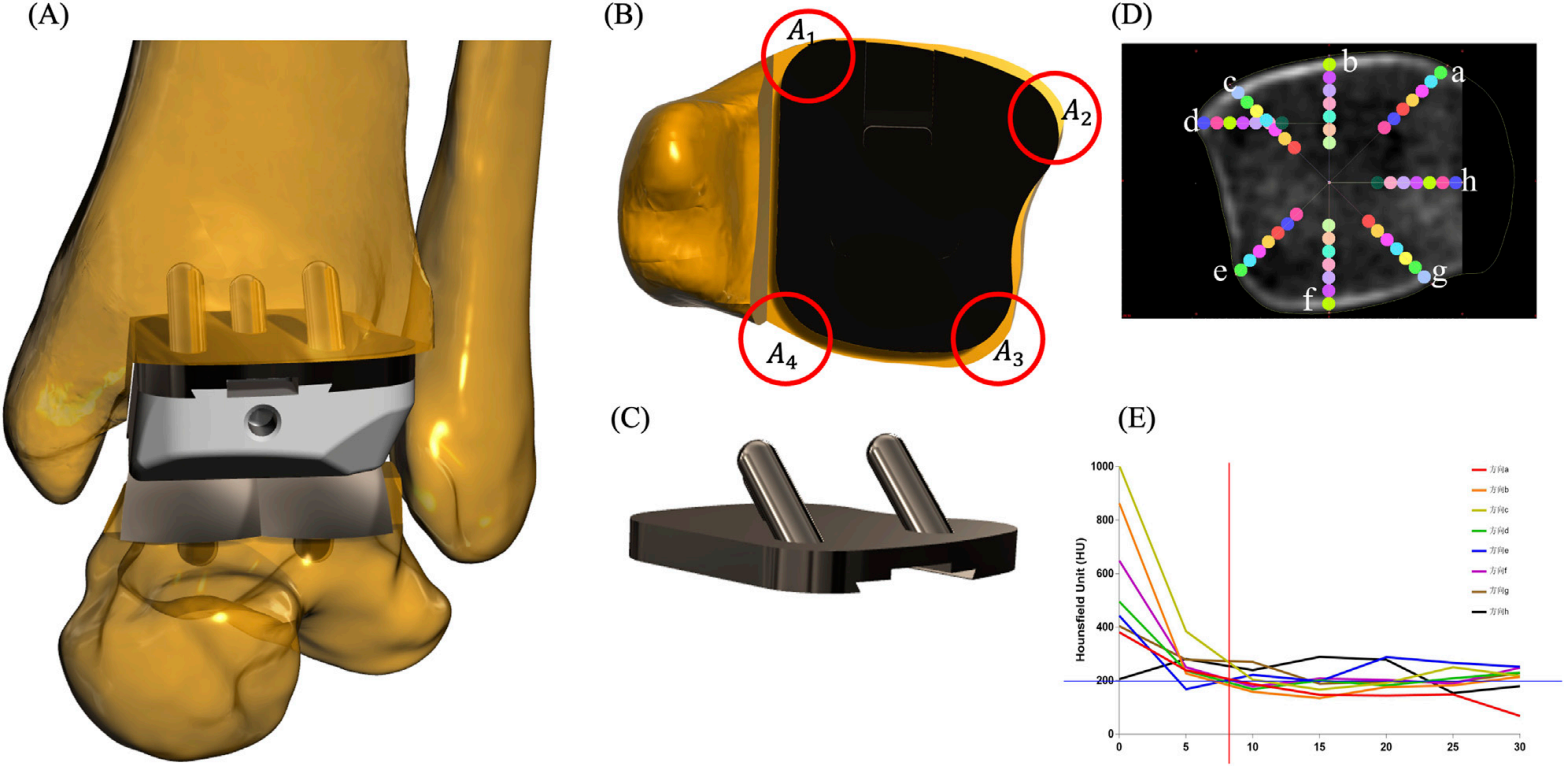
**(A)** Novel ankle implant designed by our group with maximum distal tibial coverage. **(B)** Inferior view of the distal tibial cross section and the tibial component of the ankle prosthesis (A1–A4 are common areas where prosthesis overhang occurs). **(C)** External view of the tibial component of the ankle prosthesis. **(D)** CT value analysis of the cross section 10 mm proximal to the distal tibial articular surface. **(E)** Distribution of CT values along different paths (regenerated from data published by Zhao et al., 2021).

The tibial components of several new-generation total ankle implants have been anatomically designed to support three cortices (see Figures 9A–C for illustration) and reduce fibular impingement (Gross et al., 2018; Integra, 2017). Such a design is highly related to the morphological variability of the distal tibial resection surface. Our previous studies have shown that bone density in the distal tibia decreases rapidly within 5 mm of the bony edge (Zhao et al., 2021) (Figures 9D, E), and the weight-bearing area of the distal tibia is primarily located in the peripheral cortical bone (Yu et al., 2022). Therefore, the implant should ideally reach the distal tibial bony edge to obtain the maximum support. However, oversizing in localized regions would result in overhang, which could cause bone or soft tissue impingement, especially at the four corners (A1–A4 regions) of the tibial component (Figure 9B). At the anteromedial aspect of the prosthesis (A1 region), impingement of the tibialis anterior tendon and the extensor hallucis longus tendon may occur; at the anterolateral aspect (A2 region), impingement with the extensor digitorum longus tendon or the anterior border of the fibula may occur; at the posterolateral aspect (A3 region), impingement of the peroneus longus tendon, the peroneus brevis tendon, or the anterior border of the fibula may occur; at the posteromedial aspect (A4 region), impingement of the tibialis posterior tendon may occur. These issues can cause peri-ankle pain, limited range of motion, and even surgical failure requiring implant removal.

The resection surface analysis showed substantial variation among the modes of variation in the overall group at the 10-mm resection surface. Anterior–posterior and medial–lateral distances were main sources of variation. In addition, variation frequently existed at both the anterior and posterior corners of the fibular notch in the resection surface of the distal tibia. For the tibial components of off-the-shelf total ankle implants considering maximizing cortical coverage, excessive prominence in the A2–A3 region should be avoided to prevent the prosthesis edge from overhanging the bone due to anatomical variations.

The current study has several limitations. First, the current SSM only involves CT images of healthy young participants. Future work should expand the recruitment to include the elderly population with more image modalities, such as MRI to account for the joint cartilage of the tibia (Forney et al., 2011; Nott et al., 2021). In addition, a 10-mm resection level did not fit all total ankle implant systems. Future studies should include more resection levels. Finally, the interpretation of deviation results of principal modes of variation has inherent subjectivity. Thus, more quantitative measurements should be developed in future studies to better identify the shape variability of the tibia. We should measure all actual models to obtain the maximum and minimum values of non-size-related parameters including the aspect ratio, curvature of the articular surface, medial malleolus morphology, anterior malleolus morphology, posterior malleolus morphology, fibular notch morphology, and the orientation of the distal tibial articular surface. Such data can be used to verify the authenticity of different SD models and determine each mode of variation, capturing a specific percentage of the variation. More statistical tools such as linear discriminant analysis should be included for precise and direct shape comparison.

In conclusion, SSM is an effective method of finding mean shape and principal variability. Considerable variabilities were noticed among these three groups and all principal modes of variation. Size plays a crucial role in both inter- and intra-groups, and morphological differences vary across different sizes. The male tibia has a bigger medial malleolus, anterior part of the fibular notch, and posterior malleolus. In addition, in the 10-mm resection surface of the distal tibia, variation existed along the anterior–posterior and medial–lateral directions and at both the anterior and posterior corners of the fibular notch. Such information is crucial for the implant design of the tibial components for total ankle replacement.

## Data Availability

The original contributions presented in the study are included in the article/Supplementary Material; further inquiries can be directed to the corresponding authors.
