# Supplementary material for: Statistical shape modeling of shape variability of the human distal tibia: implication for implant design of the tibial component for total ankle replacement
Source: Front Bioeng Biotechnol. 2025 Feb 27;13:1504897. doi: 10.3389/fbioe.2025.1504897 (PMC11903760; doi:10.3389/fbioe.2025.1504897)
Supplement: Supplementary file 1 [file Supplementaryfile1.docx]

**Supplementary Materials**

A sensitivity analysis of the selection on the articular surface of the distal tibia for plane fitting on one of the subjects was performed. First, based on the curvature analysis on the surface of distal tibia, manually selected the facet surface between the medial gutter and the fibular notch. Then, shrink the selection by one mesh from the edge to the center. Last, plane fitting each selection. (The sensitivity analysis was illustrated in **Figure S1）**


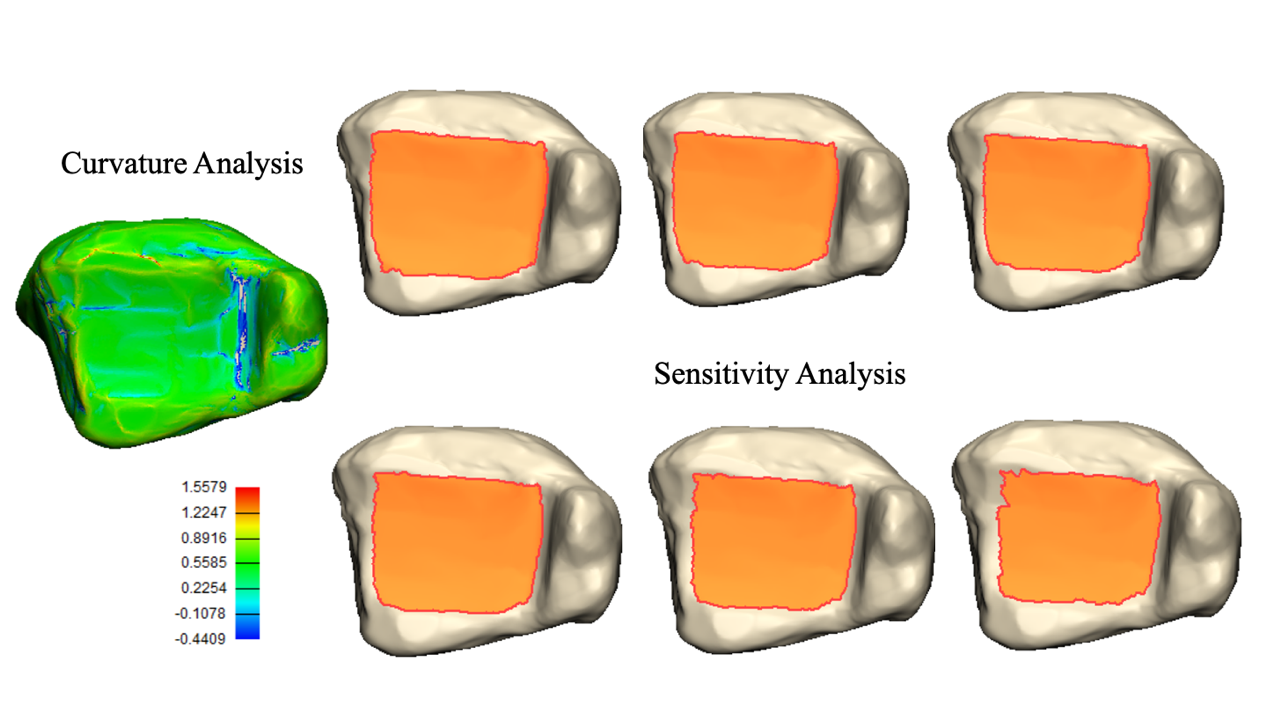


**Figure S1** an illustration of the sensitivity analysis of the area selection on the articular surface of the distal tibia for plane fitting. Shrinking the area of selection

The result for the sensitivity analysis was listed in **Table S1**. The positions of the fitting plane origins and their normal vector were not sensitive to the selection area. As the mesh size shrinks from 6230 to 4874, the maximum differences were all less than 5%. We believe current method is stable and reasonable to find the plane for distal articular surface of the tibia.

**Table S1** Sensitivity analysis of the selection on the articular surface of the distal tibia for plane fitting (the x, y and z axis of the origin and normal vector of the plane fitting the selected surfaces under different mesh sizes).

| Mesh size | 6230 | 6756 | 6230 | 5775 | 5323 | 4874 |
| --- | --- | --- | --- | --- | --- | --- |
| Origin-x | -47.0176 | -47.0379 | -47.0176 | -46.9779 | -46.8857 | -46.7208 |
| Origin-y | -178.8423 | -178.8576 | -178.8423 | -178.8299 | -178.8025 | -178.7667 |
| Origin-z | -51.0351 | -51.0733 | -51.0351 | -51.0045 | -50.9662 | -50.9201 |
| Normal-x | -0.0865 | -0.0852 | -0.0865 | -0.0879 | -0.0897 | -0.0923 |
| Normal-y | -0.0579 | -0.0575 | -0.0579 | -0.0587 | -0.0602 | -0.0611 |
| Normal-z | 0.9946 | 0.9947 | 0.9946 | 0.9944 | 0.9941 | 0.9939 |

The maximum, average, standard deviation and root mean square estimate deviation in Principal component analysis (PCA) modes 1 to 7 of the overall, female and male groups at ±3 std from the mean shape and between groups were listed in **Table S2** and **Table S3**, respectively.

**Table S2** Maximum (Max), average, standard deviation (std) and root mean square (RMS) estimate deviation in Principal component analysis (PCA) modes 1 to 7 of the overall, female and male groups at ±3 std from the mean shape.

| Group | Mode | Std | Max (Positive/ Negative) (mm) | Average (Positive/ Negative) (mm) | Standard Deviation (mm) | RMS Estimate (mm) |
| --- | --- | --- | --- | --- | --- | --- |
| Overall | 1 | -3 | 2.4699/-2.1167 | 0.9299/-0.7765 | 1.0195 | 1.0193 |
|  |  | +3 | 2.1221/-2.4700 | 0.8005/0.9199 | 1.0175 | 1.0191 |
|  | 2 | +3 | 1.4690/-1.7222 | 0.5176/-0.5689 | 0.6755 | 0.6759 |
|  |  | -3 | 1.7241/-1.4645 | 0.5956/-0.4892 | 0.6752 | 0.6833 |
|  | 3 | +3 | 2.1025/-1.1888 | 0.4452/-0.4081 | 0.5682 | 0.5720 |
|  |  | -3 | 1.1904/-2.0911 | 0.4285/-0.3872 | 0.5248 | 0.5259 |
|  | 4 | +3 | 1.1355/-1.5444 | 0.3753/-0.4426 | 0.4934 | 0.4939 |
|  |  | -3 | 1.5477/-1.1340 | 0.4538/-0.3581 | 0.4929 | 0.4966 |
|  | 5 | +3 | 1.2879/-0.9203 | 0.3841/-0.3527 | 0.4368 | 0.4392 |
|  |  | -3 | 0.9238/-1.2794 | 0.3601/-0.3709 | 0.4337 | 0.4338 |
|  | 6 | +3 | 1.0919/-1.2303 | 0.3214/-0.2687 | 0.3957 | 0.3961 |
|  |  | -3 | 1.2349/-1.0905 | 0.2943/-0.3023 | 0.4003 | 0.4009 |
|  | 7 | +3 | 0.7595/-1.1460 | 0.2765/-0.3059 | 0.3510 | 0.3528 |
|  |  | -3 | 1.1478/-0.7594 | 0.3323/-0.2735 | 0.3688 | 0.3688 |
| Female | 1 | +3 | 2.3100/-1.6242 | 0.7352/-0.5969 | 0.8415 | 0.8445 |
|  |  | -3 | 1.6252/-2.3100 | 0.6133/-0.7183 | 0.8355 | 0.8355 |
|  | 2 | +3 | 1.4272/-1.5811 | 0.5528/-0.5158 | 0.6356 | 0.6357 |
|  |  | -3 | 1.5840/-1.4167 | 0.5450/-0.5283 | 0.6374 | 0.6435 |
|  | 3 | +3 | 2.1264/-1.1229 | 0.4580/-0.4297 | 0.5734 | 0.5747 |
|  |  | -3 | 1.1330/-2.1052 | 0.4538/-0.4006 | 0.5352 | 0.5373 |
|  | 4 | +3 | 0.9695/-1.0768 | 0.3777/-0.3685 | 0.4380 | 0.4388 |
|  |  | -3 | 1.0840/-0.9648 | 0.3827/-0.3631 | 0.4394 | 0.4397 |
|  | 5 | +3 | 1.2717/-1.0353 | 0.3448/-0.2626 | 0.3924 | 0.3945 |
|  |  | -3 | 1.0360/-1.2676 | 0.2746/-0.3254 | 0.3865 | 0.3866 |
|  | 6 | +3 | 1.2226/-0.7529 | 0.3279/-0.2667 | 0.3747 | 0.3755 |
|  |  | -3 | 0.7528/-1.2174 | 0.2695/-0.3098 | 0.3618 | 0.3618 |
|  | 7 | +3 | 1.2601/-0.8108 | 0.2884/-0.2447 | 0.3497 | 0.3497 |
|  |  | -3 | 0.8117/-1.2553 | 0.2541/-0.2658 | 0.3389 | 0.3398 |
| Male | 1 | +3 | 2.6184/-1.9047 | 0.7981/-0.6736 | 0.9369 | 0.9410 |
|  |  | -3 | 1.9108/-2.6112 | 0.6886/-0.7910 | 0.9398 | 0.9405 |
|  | 2 | +3 | 1.5256/-1.4407 | 0.6293/-0.5409 | 0.6877 | 0.6980 |
|  |  | -3 | 1.4368/-1.5238 | 0.5608/-0.6057 | 0.6879 | 0.6891 |
|  | 3 | +3 | 2.0837/-1.4144 | 0.4607/-0.4019 | 0.5757 | 0.5783 |
|  |  | -3 | 1.4313/-2.0778 | 0.4210/-0.4089 | 0.5440 | 0.5443 |
|  | 4 | +3 | 1.2268/-1.2907 | 0.4178/-0.3866 | 0.5014 | 0.5090 |
|  |  | -3 | 1.2950/-1.2256 | 0.4195/-0.4089 | 0.5164 | 0.5179 |
|  | 5 | +3 | 1.5073/-1.0072 | 0.4504/-0.3545 | 0.5057 | 0.5068 |
|  |  | -3 | 1.0082/-1.5008 | 0.3640/-0.4234 | 0.4906 | 0.4908 |
|  | 6 | +3 | 1.2387/-1.0495 | 0.3673/-0.3249 | 0.4343 | 0.4344 |
|  |  | -3 | 1.0524/-1.2384 | 0.3359/-0.3417 | 0.4226 | 0.4257 |
|  | 7 | +3 | 1.0117/-1.0844 | 0.2822/-0.2937 | 0.3537 | 0.3551 |
|  |  | -3 | 1.0843/-1.0117 | 0.3109/-0.2729 | 0.3596 | 0.3596 |

**Table S3** Maximum (Max), average, standard deviation (std) and root mean square (RMS) estimate deviation among the mean models of the overall, female and male group.

|  | Max (Positive/ Negative) (mm) | Average (Positive/ Negative) (mm) | Standard Deviation (mm) | RMS Estimate (mm) |
| --- | --- | --- | --- | --- |
| Female / Overall | 0.6802/-2.9376 | 0.2007/-0.8541 | 0.6722 | 0.9318 |
| Male / Overall | 1.7150/-1.2455 | 0.8896/-0.4712 | 0.6414 | 0.9026 |
| Male / Female | 4.1466/-1.3907 | 1.6825/-0.4291 | 1.1540 | 1.7893 |
